# A Pricing Model for Groundwater Rights in Ningxia, China Based on the Fuzzy Mathematical Model

**DOI:** 10.3390/ijerph16122176

**Published:** 2019-06-19

**Authors:** Zeyu Wang, Juqin Shen, Fuhua Sun, Zhaofang Zhang, Dandan Zhang, Yizhen Jia, Kaize Zhang

**Affiliations:** Business School, Hohai University, Nanjing 211100, China; zywang0705@hhu.edu.cn (Z.W.); jqshen@hhu.edu.cn (J.S.); zhangdandan@hhu.edu.cn (D.Z.); jiayizhen@hhu.edu.cn (Y.J.); kzzhang@hhu.edu.cn (K.Z.)

**Keywords:** groundwater, water rights, fuzzy mathematical model, pricing model

## Abstract

To reduce groundwater overexploitation and alleviate water shortages, market mechanisms are introduced to allocate water rights. Scientific and reasonable pricing of groundwater rights is key to ensuring the effectiveness of the groundwater market. Because of the complexity and uncertainty of water resources, this study calculates the price of groundwater rights based on the value of water resources with an evaluation indicator system. The system includes 14 indicators developed with a fuzzy mathematics model addressing three dimensions: environment, society, and economy. The weights of the indicators are determined through the analytic network process (ANP) and the entropy method. The results show that the price of groundwater rights in Ningxia, China increased from 5.11 yuan/m^3^ to 5.73 yuan/m^3^ between 2013 and 2017; this means the price was basically stable, with a slight increase. The ratio of residents’ water fee expenditures to real disposable income also remained essentially stable, fluctuating around 0.37%, far below the normal level. These data demonstrated that the current regional water price policy does not reflect the true value of groundwater resources; there is room to increase urban water prices. Local governments need speed up water price system reforms and adopt water rights systems to optimize water resource allocations.

## 1. Introduction

Due to the decrease of the total amount of water resources caused by the uncertainty of global climate change, water resources are becoming increasingly strained. Groundwater has become an important supplement to surface water, and is attracting more and more attention. China has always been faced with water shortage problems of varying degrees, and the growing large population and uneven distribution aggravates the water resources management dilemma [1,2]. According to the statics from the ministry of water resources of the People’s Republic of China (MWRPRC), the per capita availability of water in China ranked just 108th in the world, making it one of the 21 most water-scarce countries worldwide, and has only 25% of the world’s average level.

Northwest China relies heavily on groundwater. However, with continuous development, groundwater overexploitation and groundwater pollution are becoming the main factors restricting sustainable development. According to MWRPRC statistics, average annual groundwater overexploitation in China increased from 10 billion m^3^/a in the 1980s to 22.8 billion m^3^/a in the 2000s; the average area of groundwater overexploitation has expanded from 56,000 km^2^ to 180,000 km^2^ during the same period. The continuous expansion of groundwater exploitation has led to the formation of regional water level depression funnels and serious geological problems, such as salinization, land subsidence, ground fissures, and karst collapses [3].

For example, Ningxia, a province located in the arid area of Northwest China, is suffering problems caused by groundwater overexploitation. According to the Ningxia Water Resources Bulletin, in 2017, there were five groundwater overexploitation areas in Ningxia, with total area of 741 km^2^. Specifically, Yinchuan City has one overexploitation area of 294 km^2^ and Shizuishan City has four overexploitation areas with a total area of 447 km^2^. The total groundwater overexploitation in Ningxia is 28.38 million m^3^. The average groundwater depth has increased by about 1 m compared with the level in 1980, and the consequent geological disasters such as land subsidence and karst collapses have caused serious economic losses and social crises. These have directly or indirectly restricted the sustainable development of regional economies. The effective allocation and rational use of groundwater resources is critical to harmoniously unify ecological and social development.

In order to control overexploitation, the government has implemented legislation such as the Water Law of PRC, Regulations on Administration of Water Abstraction Licensing and Collection of Water Resources Charges, Regulations on the Development and Utilization of Urban Groundwater and the Law on the Prevention and Control of Water Pollution. The contents of these laws and regulations mainly include the following aspects: First, delimit the groundwater overexploitation area. Second, strictly control the groundwater exploitation. Third, strengthen well management in groundwater overexploitation areas. Fourth, artificially recharge groundwater.

Although the legislation has reduced groundwater overexploitation to a certain extent, it has little effect on the low efficiency of groundwater utilization. A new method of water resources allocation needs to be introduced. Water trading markets is a good way to improve the efficiency of water resources [4,5,6,7,8], which will be helpful to promote the areas of low utilization rate of groundwater flowing to the areas with high utilization rate of groundwater [9,10]. For this reason, China, Australia, and South Asia have some kind of groundwater trading markets [11,12,13,14,15]. However, compared with the increasingly mature and perfect surface water market in these areas [16], the incompleteness of groundwater price mechanism restricts the effectiveness of the groundwater market [17]. Scientific and reasonable groundwater pricing urgently needs to be studied for the optimization of allocating groundwater resources [18]. 

There are many classical surface water pricing methods, such as the shadow price method [19], the supply and demand price model [20], cost pricing method [21], real option method [22], game method [23], and principal-agent method [24]. Many pricing factors have been proposed for water rights, including resource utilization efficiency and cost [25], ecological sustainability [26], seasonality [27], and water users and market [28]. However, these achievements cannot be directly applied to groundwater pricing, because of the difficulty of transporting groundwater resources across regions [29] and the complexity of groundwater recharge compared with surface water [30], which makes it difficult to use conventional mathematical models in pricing. In addition, current studies on groundwater rights pricing method consider just one single factor [31,32,33,34], ignoring the fact that the pricing of groundwater rights is a complex system of interactions and couplings between natural systems, economic systems, and social systems.

To fill these gaps, we propose a scientific and feasible pricing method for groundwater using fuzzy mathematics [35,36,37,38,39]. This paper uses fuzzy mathematics combined with the analytic network process (ANP) and entropy method to estimate the fuzzy value of groundwater rights, which is multiplied with the price vector in the price calculation model to obtain the groundwater rights price. It is worth mentioning that unlike the traditional method, this study selects environment, society, and economy as the pricing indicators and calculates each indicator’s weight. As a result, the study generates dynamic trends of groundwater rights pricing in Ningxia from 2013 to 2017 which is conducive to improving the utilization efficiency of groundwater and the sustainable development of water resources in China. This paper is organized into four parts. Section 1 is the introduction. In Section 2, the materials and methods are introduced, including the research area and the pricing method. Section 3 details the statistical data, the calculation results of the model, and a discussion of the groundwater rights price. Finally, the conclusions are provided in Section 4.

## 2. Materials and Methods

### 2.1. Research Area

**Figure 1:**
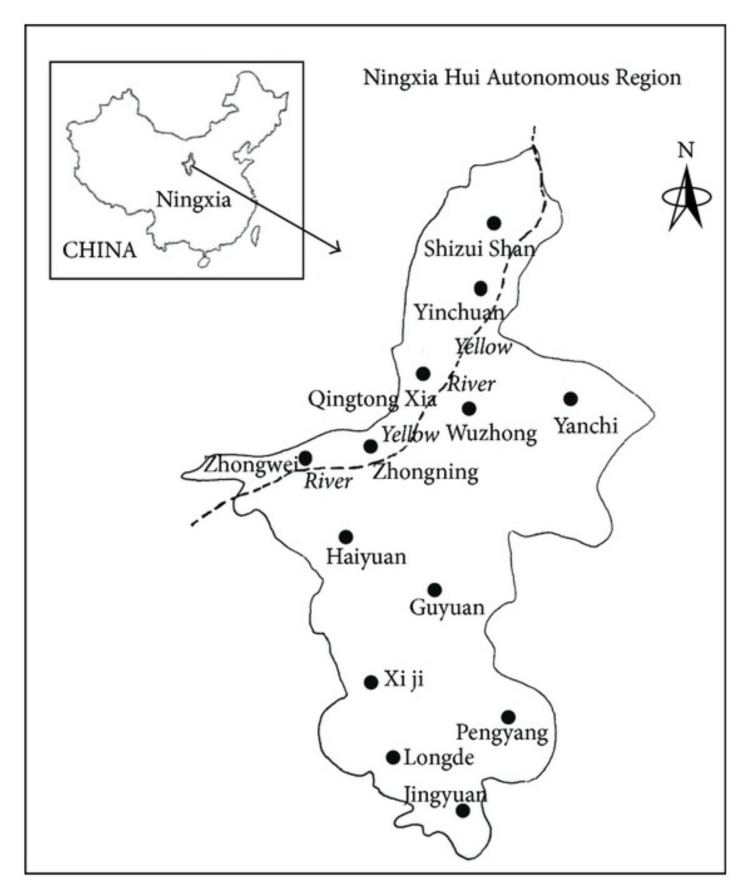
Location and region map of Ningxia.

Ningxia is short for Ningxia Hui Autonomous Region, which is located in the middle and upper reaches of the Yellow River in northwest China (35°14′N–39°23′N, 104°17′E–107°39′E) and adjacent to Gansu Province, Inner Mongolia Autonomous Region, and Shaanxi Province. The northern part belongs to the Inner Mongolia Plateau, surrounded by the Tenggeli, Ulanbuhe, and Maowusu deserts. The southern part belongs to the Loess Plateau. The total area of the region is 664,400 km^2^, and it has five prefecture-level cities. In 2017, the permanent population was 681,790. Figure 1 shows a location and regional map of Ningxia [40]. The per capita GDP of Ningxia in 2017 was 50,765 yuan, ranking 15th of all the provinces in China. Ningxia’s economy is very extensive and its water use efficiency is very low. In 2017, the per 10^4^-yuan-GDP water consumption is 191.95 m^3^, ranking 4th of all the provinces in China.

Ningxia province is one of China’s provinces with the most significant water resource shortages, which hinders the further development of the local economy. In 2017, the total annual precipitation for Ningxia was 332 mm, which was 17.177 billion m^3^ in terms of total precipitation (ranking 3rd from the bottom). Ningxia’s total water resources were quantified at 1.077 billion m^3^ (ranking last). Of all the water resources, surface water resource quantities accounted for 865.3 million m^3^ (ranking last), groundwater resource quantities were 1.933 billion m^3^ (ranking 3rd from the bottom), the double counting amount between surface water and groundwater was 1.721 billion m^3^, and total water consumption was 6.606 billion m^3^. Table 1 lists the groundwater resources in Ningxia according to “Ningxia Water Resources Bulletin 2017” [41]. The data are estimates from the Ningxia Water Conservancy based on the groundwater hydrological data measured at monitoring stations.

The shortage of groundwater sources in Ningxia has seriously affected local development, calling for attention of both the government and the general public. Therefore, how to improve the utilization efficiency of groundwater in Ningxia and reduce overexploitation are the key problems. However, at present, most of the research on the water resource governance in Ningxia focuses on fields such as water distribution [42,43], benefits of water rights transfer [44], and water resources sustainable utilization [45]. However, there is a lack of research on groundwater rights pricing. As a result, the water price is unscientific and the government’s pricing policy lacks appropriate support. Therefore, to develop a reasonable pricing scheme for water resources by drawing on the existing Ningxia water rights trading market is the focus of this paper. Our findings might be helpful for other similar provinces.

### 2.2. Data Sources

Based on data operability and availability, the data used in this study were derived from the “Ningxia Water Resources Bulletin 2013–2017” [41], “Ningxia Statistical Yearbook 2013–2017” [46], “Ningxia Autonomous Region Statistical Bulletin of National Economic and Social Development 2013–2017” [47], “Monthly Report of Groundwater Regime in China January 2013–December 2017” [48], and “China Tap Water Price 2013–2017” [49].

### 2.3. Methods

Groundwater rights are defined as the property rights associated with groundwater resources. As demonstrated above, a reasonable price for groundwater rights should be based on the value of groundwater resources in the government-led primary market. This value can be measured by a fuzzy synthetic evaluation model. The model used in this study to quantitatively evaluate the price of groundwater rights in Ningxia can be divided into two parts: the groundwater rights fuzzy evaluation model and the price calculation model. In the groundwater rights fuzzy evaluation model, we calculate the fuzzy value of groundwater rights, which is combined with the price vector to calculate the price.

#### 2.3.1. Groundwater Rights Fuzzy Evaluation Model

In this model, the fuzzy value is estimated with the synthetic weight vector of pricing indicators and the evaluation matrix. The first step is to select pricing factors. This paper proposes three kinds of factors: environment, society, and economy. The fuzzy value of groundwater is expressed in the following equation:

(1) $$V=f\left(X_{1},X_{2},X_{3},\cdots ,X_{n}\right)$$

where V is the value of groundwater rights and $X_{1},X_{2},X_{3},\cdots ,X_{n}$ are the selected indicators characterizing the factors affecting the value of groundwater rights. The selection of pricing indicators affects the effectiveness of groundwater rights price. A reasonable price for groundwater rights need have three main functions [50]:

- Save groundwater resources and improve the utilization rate of groundwater resources
- Protect groundwater resources and the water environment
- Encourage different funds to invest in groundwater resources’ development, utilization, allocation, conservation, and protection.

Based on this, referring to relevant experience related to water rights pricing [51], indicator selection should consider the following principles:

(1) Sustainable development. Scientific and reasonable prices should coordinate the interests of governments, enterprises, and water users; coordinate ecological, economic, and social benefits; coordinate contemporary and future generation benefits; and ultimately realize the fairness and sustainability of water resources utilization in space and time.
(2) Efficient utilization. The insufficient supply of groundwater makes it difficult to meet all needs. Therefore, from the economic perspective, we should pursue the best use of water under the guidance of price tools. This allows groundwater to be allocated to places with high utilization efficiency and to create more value. This involves striving to maximize the utilization of groundwater resources.
(3) Fairness. Water resources are necessary for human production and life. As such, it is necessary to ensure the basic needs of all people, to ensure the fair allocation of groundwater in different regions and across different industries and groups. It is particularly important to ensure the water rights of vulnerable groups.

The selection of indicators is detailed in Section 3.1. After the factors are determined, the domain U is set for the groundwater pricing indicators. The U domain is expressed by Equation (2).

(2) $$M=\left\{X_{1},X_{2},X_{3},\cdots ,X_{n}\right\}$$

This equation means that the domain of the model is the pricing indicators of groundwater rights. The evaluation vector of pricing indicators is shown in Equation (3):

(3) H={High, Relatively High,Common, Relatively Low, Low}

where H is the evaluation vector. This equation means that all the pricing indicators are evaluated into five grades. Equations (2) and (3) are used as presupposition. By setting the synthetic weight vector and the evaluation matrix of pricing indicators, Equation (1) is transformed into Equation (4):

*V* = *W*·*R* (4)

The fuzzy value of groundwater rights can be estimated by this equation. In this formula, V is the value of groundwater rights; W is the synthetic weight vector of pricing indicators; and R is the evaluation matrix, shown as Equation (5):

(5) $$R=\left|\begin{array}{c} R_{1}\\ R_{2}\\ R_{3}\\ \vdots \\ R_{n} \end{array}\right|=\left[\begin{array}{ccccc} r_{11} & r_{12} & r_{13} & r_{14} & r_{15}\\ r_{21} & r_{22} & r_{23} & r_{24} & r_{25}\\ r_{31} & r_{32} & r_{33} & r_{34} & r_{35}\\ \vdots & \vdots & \vdots & \vdots & \vdots \\ r_{n1} & r_{n2} & r_{n3} & r_{n4} & r_{n5} \end{array}\right]$$

In this expression, $r_{ij}\left(i=1,\;2,\;3,\cdots ,\;n;\;j=1,\;2,\;3,\;4,\;5\right)$ is the membership degree of indicator *i* to evaluation factor grade j. This study chose the ascending (descending) semitrapezoidal distribution and established a linear membership function [52] to calculate the membership degree of each indicator in matrix R to different evaluation grades in vector H. That is,

When  j=1,

(6) $$r_{i1}=\{\begin{array}{cc} 1 & x_{i}\leq x_{i1}\\ \frac{x_{i2}-x_{i}}{x_{i2}-x_{i1}} & x_{i1}<x_{i}<x_{i2}\\ 0 & x_{i}\geq x_{i2} \end{array}$$

When j=2, 3, 4,

(7) $$r_{ij}=\{\begin{array}{cc} 0 & x_{i}\leq x_{i,j-1}\\ \frac{x_{i}-x_{i,j-1}}{x_{i,j-1}-x_{i,j}} & x_{i,j-1}<x_{i}<x_{ij}\\ \frac{x_{i,j+1}-x_{i}}{x_{i,j+1}-x_{i,j}} & x_{i,j}<x_{i}<x_{i,j+1}\\ 0 & x_{i}\geq x_{i,j+1} \end{array}$$

When j=5,

(8) $$r_{i5}=\{\begin{array}{cc} 0 & x_{i}\leq x_{i4}\\ \frac{x_{i}-x_{i4}}{x_{i5}-x_{i4}} & x_{i4}<x_{i}<x_{i5}\\ 1 & x_{i}\geq x_{i5} \end{array}$$

where $x_{i}$ is the actual value of evaluation indicator i, and $x_{i,j-1}$, $x_{i,j}$, $x_{i,j+1}$ is the criterion value of the two adjacent levels of the evaluation indicator. With Equations (6)–(8), the evaluation matrix R is calculated. To achieve the value of groundwater rights, the next step is to calculate the synthetic weight vector of indicators (W) according to Equation (4). The calculation process is detailed in Section 2.3.2.

#### 2.3.2. Factor Weights Calculation Method

Based on the importance of each evaluation indicator, a corresponding weight is assigned. ANP is a commonly used decision-making tool. The weight of the model is obtained by comparing the factors in pairs, combining the comparison results with fuzzy mathematics, and constructing a hyper matrix [53]. Considering the interlocking interaction between pricing factors, ANP is a suitable tool. However, the ANP model is easily influenced by researcher subjectivity, leading to errors in the results. Therefore, the ANP model is often combined with an entropy weight method, and objective coefficients are introduced to modify the results [54,55]. In this paper, the synthetic weights are also obtained by combining these two models.

The ANP model works by constructing supermatrices. With the software “Super Decisions”, the supermatrices can be constructed by the following steps:

(1) According to the network hierarchy, the pricing indicators were compared pairwise. Setting a certain indicator as the criterion, the relative influence intensity of the other elements influencing the indicator was evaluated. Generally, the magnitude of influence was expressed with the numbers from 1 to 9.
(2) Construct the unweighted supermatrix. The element in the matrix, for example, ω
ij, was the degree of influence of the element i on the element j.
(3) Similarly, the influence vectors between clusters were obtained by comparing two clusters with each other. The unweighted supermatrix was multiplied by the corresponding weight to obtain the weighted supermatrix. The power operation was applied to the weighted supermatrix, so that the power exponent tended to infinity. When every column element no longer changed, the limit matrix was obtained. The limit matrix shows the quantitative influence of all the indicators on the price, namely, the weights of indicators.

As mentioned above, this result is so subjective that it needs to be combined with the result of the entropy weight method. The indicator initial value matrix was set as U, in which the element *μ_ij_* represented the initial number value of indicator i for item j. Based on matrix U, another weight was calculated with the entropy method through the following steps:

(1) Data standardization. This paper used 0-1 standardization. The formula is:
  (9) $$\mu_{ij}^{\prime }=\{\begin{array}{c} \frac{\mu_{ij}-min\left(\mu_{ij}\right)}{\max\left(\mu_{ij}\right)-min\left(\mu_{ij}\right)},\mu_{ij}\;is\;positive\\ \frac{\max\left(\mu_{ij}\right)-\mu_{ij}}{\max\left(\mu_{ij}\right)-min\left(\mu_{ij}\right)},\mu_{ij}\;is\;negative \end{array}$$
  where $\mu_{ij}$ is the element of matrix U, and $\mu_{ij}^{\prime }$ is the standardized element corresponding to $\mu_{ij}$.
(2) Data normalization. The formula is:
  (10) $$d_{ij}=\frac{\mu_{ij}^{\prime }}{\sum_{i=1}^{n}\sum_{j=1}^{m}\mu_{ij}^{\prime }}$$
  where $d_{ij}$ is the normalized element corresponding to $\mu_{ij}^{\prime }$ n is the total of the indicators; and m is the total of items in the research.
(3) Calculate the entropy of each indicator. The entropy of indicator i is calculated as Equation (11)–(12).
  (11) $$E_{i}=-\frac{1}{lnm}\overset{m}{\underset{j=1}{\sum }}\frac{1+d_{ij}}{\sum_{j=1}^{m}\left(1+d_{ij}\right)}ln\left[\frac{1+d_{ij}}{\sum_{j=1}^{m}\left(1+d_{ij}\right)}\right]$$
  (12) $$h_{i}=\frac{1-E_{i}}{\sum_{i=1}^{n}\left(1-E_{i}\right)}$$
  where $h_{i}$ is the entropy and weight of indicator *i*.

The synthetic weight of indicator is calculated with these two weights. Specifically, setting the weight derived from ANP model as the subjective weight of indicator *i*, with the entropy of indicator *i* as the objective weight, a synthetic weight of indicator *i* can be obtained with Equation (13) [56]:

(13) $$\omega^{i}=\frac{\omega_{1}{}^{i}\omega_{2}{}^{i}}{\sum_{i=1}^{n}\omega_{1}{}^{i}\omega_{2}{}^{i}}$$

where $\omega_{1}{}^{i}$ is the subjective weight, $\omega_{2}{}^{i}$ is the objective weight ($\omega_{2}{}^{i}=h_{i})$, and $\omega^{i}$ is the synthetic weight of indicator i. with the evaluation matrix R and the synthetic weight vector W formed with $\omega^{i}$, the value of groundwater rights V is calculated.

#### 2.3.3. Price Calculation Model

The next step is to determine the price vector, which is usually obtained by equally dividing the range of water prices corresponding to the evaluation grades, based on the actual situation. The lower limit of groundwater price is 0, and the upper limit of groundwater price is affordability for water users. Traditionally, the affordability for water users is calculated based on the proportion of an urban resident’s living water expenditure to their income. This is called the water rate affordability index [57]. Similarly, the affordability is calculated as Equation (14):

(14) P=A×E/Q−C−D−T

In this formula, P is the upper limit of groundwater price; A is the water rate affordability indicator; E is the per capita disposable income of residents; Q is the annual per capita water consumption (In fact, per capita water requirement is better. However, it is difficult to accurately quantify the per capita water requirement due to the difference of water resources endowment and water use habits in subordinate regions. Referring to similar literature, this paper selects per capita water consumption as an alternative); C is the water supply cost and normal profit; D is the sewage treatment fee; and T is the water resource fee. This fee is charged in China due to the scarcity of water resources and the upfront protection input of the government. This study selected price affordability as a key factor, for which there is little difference between surface water and groundwater. As such, this equation does not distinguish between these two kinds of water resources. The water rate affordability index is usually set by researchers depending on the region being studied. Research by the World Bank shows that 5% is the maximum affordability index for developing countries. Most scholars believe that 3–5% is a reasonable ratio; as such, 3% is a realistic and feasible ratio for developing countries [58].

Also, the water rate affordability index can be evaluated with the proportion of water fee expenditure to per capita real disposable income, as expressed in Equation (15):

(15) A=CW/AI

where A is the water rate affordability index; CW is the per capita water fee expenditure; and AI is the per capita real disposable income. The per capita disposable income excludes price effects. By comparing the calculated result with the set ratio, whether the current water rate is high or not can be judged.

Then, the groundwater rights price vector S is obtained as Equation (16):

(16) $$S={\left[P,\;0.75P,\;0.5P,\;0.25P,\;0\right]}^{T}$$

This vector measures the affordability of groundwater rights prices. The five elements in the vector correspond to the five grades in the evaluation vector. Finally, the price of groundwater rights can be calculated by combining the fuzzy value V and the price vector S as Equation (17):

(17) GP=(1+α)V·S,

where, GP is the price of groundwater rights, S is the price vector, V is the value of groundwater rights, and “α” is a parameter reflecting government regulation. These include extra fees for highly polluted industries, prohibition of trade in groundwater reserves, and support for vulnerable industries such as agriculture.

## 3. Results and Discussion

### 3.1. Evaluation Indicators and Actual Value

Based on the principles in Section 2.3.1 and referring to studies on the evaluation of other resources by ANP [59,60,61], three kinds of indicators were selected for this study: environment, society, and economy. Data of all the indicators were obtained from the government bulletin in Section 2.2, which can be seen in Table 2.

Environmental indicators characterize factors associated with the groundwater environment. These indicators are critical, because one of the major objectives of water pricing is to protect the groundwater environment. Specifically, for groundwater resources, the main objective is to prevent pollution and reduce overexploitation. Groundwater quality grade, per capita groundwater resources, groundwater resources per unit area, average depth of shallow groundwater, and the proportion of overexploited groundwater area were selected as indicators to reflect the environmental factors impacting groundwater rights pricing. The groundwater quality grade is an indicator reflecting groundwater quality, defined based on Specification GB/T14848-2017, “Standard for Groundwater Quality”. The better the water quality is, the higher the water price is. Per capita groundwater resources and groundwater resources per unit area reflect the degree of regional scarcity in groundwater resources, from the perspectives of population and region. In areas with abundant groundwater resources, the price of water rights is low. Conversely, in areas where groundwater is relatively scarce, if groundwater rights are tradable (in certain areas, such as protected areas and reserved areas of groundwater, the policy may prohibit or restrict groundwater exploitation and groundwater right transactions. These areas were excluded from the scope of the study), the price of water rights is high. The average depth of shallow groundwater reflects the groundwater storage in plain areas. The deeper the average shallow groundwater depth is, the more difficult it is to exploit the groundwater. As a result, the price of groundwater rights is higher.

Social indicators characterize factors reflecting groundwater demand, and mainly include population and water withdrawal. Population density, per capita groundwater withdrawal, agricultural water withdrawal, industrial water withdrawal, and withdrawal for residential living use were selected as indicators to reflect the social factors impacting groundwater right pricing. Population density is an indicator reflecting the local population size. In general, the higher the population density is, the more water consumption there is, and the higher the water price is. The per capita groundwater withdrawal is an indicator reflecting the overall market demand. The larger per capita groundwater consumption will raise the demand for groundwater rights, which eventually leads the higher the price of groundwater rights. Therefore, agricultural groundwater withdrawal, industrial groundwater withdrawal, and the withdrawal of groundwater for residential living were selected to reflect groundwater demand of different industries.

Economic indicators characterize macroeconomic conditions that impact water prices. Per capita GDP, per 10^4^-yuan-GDP water consumption, the proportion of the groundwater supply, and wastewater discharge were selected as indicators to reflect the economic factors impacting groundwater right prices. Per capita GDP is a common indicator reflecting regional economic level. The reason why we use this indicator is that could have a positive on groundwater price. In general, a higher regional economy will lead to an increase in per capita water consumption, which will be reflected in an increase of groundwater price. The per 10^4^-yuan-GDP water consumption is an indicator reflecting the water use efficiency of regional economic sectors. Similar to per capita GDP, there is a positive correlation between water consumption and water price. If the value of water consumption per unit GDP is lower, the higher water use efficiency will decrease the groundwater price. The proportion of groundwater supply (groundwater supply/total water supply) reflects the degree of reliance on groundwater in the region, compared with surface water. The higher the proportion of groundwater supply is, the higher the price of groundwater rights is. Lastly, the wastewater discharge is an indicator reflecting the negative externality of the economy. It is worth noting that groundwater trade needs to account for environmental protection requirements. Hence, when the groundwater is used in industries with serious pollution, an additional price needs to be charged. Table 3 lists the indicators and the data.

### 3.2. Evaluation Criteria of Indicators

The evaluation criteria of groundwater rights value were divided into five grades as shown in Equation (3): High, Relatively High, Common, Relatively Low, and Low. All grades are qualified by referring to the annual average data of every provinces in China [62,63]. Specifically, the upper and lower limits are determined according to the annual average provincial data. Then, the criterion values can be calculated by the interval arithmetic method after the highest criterion and lowest criterion are set.

In addition, there is no quantitative statistics of the indicator “groundwater quality grade” in the bulletin, which means the provincial data of this indicator cannot be averaged. Thus, the evaluation criterion of this indicator had to be determined according to classification in the national standard of groundwater quality (Specification GB/T14848-2017, “Standard for Groundwater Quality”).

There are two kinds of indicators: positive indicators and negative indicators. For positive indicators, the bigger the number is, the higher the value it reflects. In contrast, for negative indicators, the greater the number is, the lower the value it reflects, and vice versa, as shown in Table 4.

### 3.3. The Network Structure and Weights

**Figure 2:**
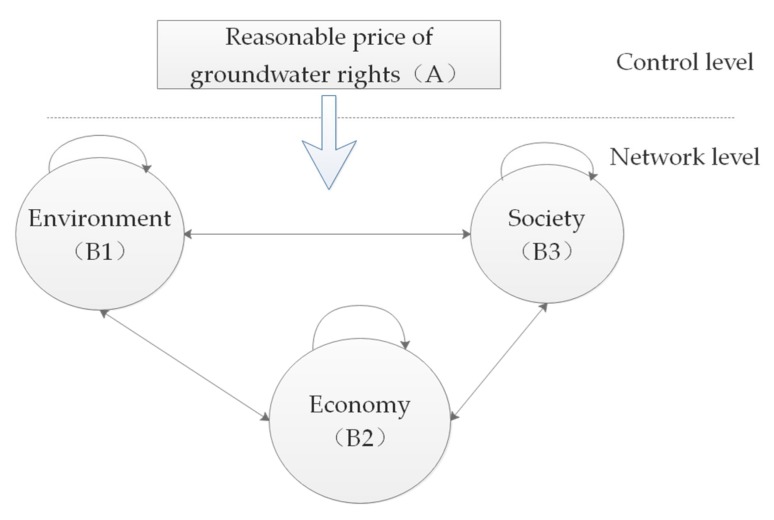
The network structure of clusters in the ANP model for groundwater rights pricing.

As mentioned above, according to the ANP model, the indicators form a network structure which makes the model more realistic than models such as AHP. Figure 2 shows the network structure associated with this research. Each node in the network represents a cluster of indicators. Considering that there is a certain correlation between each cluster of indicators, the change of each indicator may cause the change of another indicator. There are 3 clusters in the network: environment, society, and economy. Interactions are represented by “→”, and “A→B” means that cluster (or factor) A is affected by the cluster (or element) B. 

Based on this structure, the indicators are compared pairwise to obtain the subjective weights following the steps in Section 2.3.2. Then, the objective weights of all the indicators are calculated and combined with the entropy method according to Equations (9)–(12). In these equations, the values of parameters n and m in the equations are determined according to the set of this study: n is the total number of indicators and m is the total number of items, namely, *n* = 14 and m = 5. Table 5 shows the calculated result of the weights.

By combining these two weights, the synthetic weights are calculated as Equation (13). The synthetic weight vector W is:

W = [0.0255, 0.2583, 0.2030, 0.0150, 0.0107, 0.1317, 0.0191, 0.0502, 0.0281, 0.0056, 0.1765, 0.0782, 0.0044, 0.0190](18)

### 3.4. Fuzzy Synthetic Evaluation of Groundwater Rights Price

The evaluation matrix R is constructed from Equations (5)–(8). In addition, as the indicator “Groundwater quality grade” could not be quantified directly, the proportion of groundwater resources with different grades was applied to calculate the membership degree of corresponding evaluation grades, applying the principle of fuzzy statistics.

For example, the fuzzy synthetic evaluation matrix of groundwater rights for 2013 was determined as follows:

(19) $$R=|\begin{array}{c} R_{1}\\ R_{2}\\ R_{3}\\ R_{4}\\ R_{5}\\ R_{6}\\ R_{7}\\ R_{8}\\ R_{9}\\ R_{10}\\ R_{11}\\ R_{12}\\ R_{13}\\ R_{14} \end{array}|=[\begin{array}{ccccc} 0 & 1 & 0 & 0 & 0\\ 0.6809 & 0.3191 & 0 & 0 & 0\\ 0.3243 & 0.6757 & 0 & 0 & 0\\ 0 & 0 & 0 & 0.1848 & 0.8152\\ 0 & 0 & 0 & 0.5720 & 0.4280\\ 0 & 0 & 0 & 0 & 1\\ 0 & 0 & 0.7000 & 0.3000 & 0\\ 0 & 0 & 0 & 0 & 1\\ 0 & 0 & 0 & 0.6104 & 0.3896\\ 0 & 0 & 0 & 0.5180 & 0.4820\\ 0 & 0 & 0 & 0.5486 & 0.4514\\ 1 & 0 & 0 & 0 & 0\\ 0 & 0 & 0 & 0.4750 & 0.5250\\ 0 & 0 & 0 & 0 & 1 \end{array}]$$

As a result, the value of groundwater rights V in 2013 can be evaluated according to Equation (4), using the calculation results from Equations (18)–(19):

V=W·R=[0.3119, 0.2451, 0.0134, 0.1337, 0.3135]

The value of groundwater rights V in other years can be calculated with the same approach.

### 3.5. Calculation of Groundwater Rights Price

The price of groundwater rights is determined by Equations (14)–(17). The numerical results of the necessary parameters can be determined by referring to the government gazette listed in Section 2.2. For the water rate affordability indicator, this paper selects 2% as a sound ratio, based on the actual situation of Ningxia. In addition, the tap water price in China varies for different users. The data related to urban resident water use were adopted in this study, as urban residents have lower affordability levels with respect to water prices than nonresidents and special industries. “Water supply cost and normal profit” is difficult to directly count; in China, this parameter is generally calculated as the residential tap water price reduces the sewage treatment fee and water resources fee. Parameter α was determined through interviews with experts. Experts believed that Ningxia has a significant water resources shortage and needs to appropriately raise the price of groundwater rights. Ningxia’s economy is underdeveloped, and an extravagant price would create burden for residents. After comprehensive consideration, the value of the parameter was set at 4% in this study.

For example, for 2013, the upper limit of the groundwater rights price in Ningxia can be calculated as Equation (14):

P=A×E/Q−C−D−T=2%×14565.78/26.28−0.90−0.85−0.31=9.03

Then, the groundwater rights price vector S is calculated as Equation (16):

$$S={\left[P,\;0.75P,\;0.5P,\;0.25P,\;0\right]}^{T}={\left[9.03,\;6.77,\;4.52,\;2.26,0\right]}^{T}$$

Finally, the price of groundwater rights is calculated as Equation (17):

$$\mathrm{GP}=(1+\alpha )V\cdot S=(1+4\%)\times [0.3199,\;0.2451,\;0.0134,\;0.1337,\;0.3135]\times [9.03,\;6.77,\;4.52,\;{2.26,0]}^{T}=5.11$$

Table 6 shows the parameter values and final calculation results of all the years in the study period.

Table 6 shows that the price of groundwater rights in Ningxia increased from 5.11 yuan/m^3^ to 5.73 yuan/m^3^ between 2013 and 2017, which is basically stable with a slight increase. This calculation result is much higher than the current actual water price (around 2.00 yuan/m3).

In addition, the ratio of residents’ water fee expenditure to real disposable income was also basically stable, fluctuating around 0.37%. This figure is lower than the domestic average and the international standard. Previous research indicates that different ratios will have different impacts on residents’ psychology and on the bearing capacity of water usage. A ratio of 1% or less had little impact on the psychology of residential water use. As the ratio increases, so does the impact on residents. When the water fee expenditure exceeds 2% of personal disposable income, it has a stronger impact and causes people to begin to care about water consumption. When the water fee expenditure accounts for 3–5% of personal disposable income, the impact is sufficient for residents to pay more attention to water usage and the economical utilization of water resources. When the ratio is 5% or more, the influence is sufficiently strong to make residents think about water reuse [64]. In other words, Ningxia’s water fee is too low to incentivize residents to save water, which is unscientific and unreasonable in such an arid area. This shows that the price policy for water resources in Ningxia has significant room for improvement.

The low price of water in Ningxia is mainly caused by both historical reasons and practical reasons. Because of the backward economic development of Ningxia, the lower water price has helped to maintain social stability. From the perspective of practical reasons, the lack of relatively advanced water management policies and water price policies leads to the low price of water resources. Hence, the Ningxia’s pricing policies are only based on economic benefits without considering environmental factors. To make matters worse, the price reform has been slow because people have become accustomed to low water prices.

In Ningxia, approximately 89% of residents rely on groundwater for living. A long-term low water price does not serve to protect groundwater resources. Although the government has adopted methods such as ladder pricing to explore reasonable water pricing, the calculations show that the effect is not significant. At present, groundwater in Ningxia is seriously overexploited due to lack of water resources. As a result, the regional economic development is restricted, and people’s lives are adversely affected. Conflicts arising from water resources occur from time to time, and sustainable development is gravely impeded. Therefore, Ningxia’s problem, common in many districts of China, needs to be solved as soon as possible.

As mentioned above, the Coase theorem suggests that by clearly defining groundwater rights and establishing a groundwater rights market to raise the price to normal levels [65], the government can effectively incentivize residents to save water and solve certain water shortage problems [66,67]. At present, the implementation of this policy faces two major obstacles. First, the relevant laws and regulations are not perfect. This is because studies on water rights and water rights trading started late in China, and the experience of other countries is not fully compatible. This problem can only be solved by practicing while studying. Another issue is public acceptance. As mentioned above, people have become accustomed to low water prices. Therefore, by introducing water rights, raising water prices may be opposed by the public, even if the price is still within the range of affordability. Therefore, when raising water prices, it is necessary to conduct hearings to fully communicate with the public, and gradually raise water prices by stages and by industry. Moreover, because of the characteristics of groundwater itself, it is necessary to adopt differential pricing among industrial water, agricultural water, and residential water.

While the pricing of groundwater rights in this paper is based on the value of groundwater resources, pricing methods and theories related to water rights are not unique. As mentioned above, the study adopted a pricing method based on the cost of groundwater supply or welfare water prices as a quasipublic product. In China, different regions adopt different pricing methods according to their own degree of economic development and groundwater resources. The pricing of groundwater rights is quite individualized and regional.

## 4. Conclusions and Recommendations

This study focused on Ningxia, China, and calculated the price of groundwater rights for the region based on the value of groundwater resources. An evaluation indicator system with 14 indicators was developed based on a fuzzy mathematics model with three dimensions: environment, society, and economy. The weight of each indicator was determined with the ANP and entropy method. This process generated pricing for groundwater rights in Ningxia from 2013 to 2017. According to the calculation results, the price of groundwater rights was basically stable a slight increase from 5.11 yuan/m^3^ to 5.73 yuan/m^3^ from 2013 to 2017. This pricing was approximately twice the actual water price (around 2.00 yuan/m^3^) at that time. Also, during this period, water fee expenditures accounted for approximately 0.37% of residents’ disposable income, which was lower than the set value of 2%. That means that the current water price policy of Ningxia underestimates the current water value, and there is still much room for water price to rise. 

Ningxia is a province short of water resources where the contradiction between supply and demand of water resources is very sharp. It is particularly important to optimize the allocation of limited water resources and reasonable water price is the basis of realizing optimal allocation. In Ningxia, the long-term low water price policy has resulted in low water costs, thus the water users waste water resources, which aggravates the water supply and demand crisis and aggravates water pollution.

After the formation of the water price mechanism that meets the requirements of the market, the local government can make full use of price leverage to adjust groundwater demand, allocate groundwater resources, and promote water saving. Water price leverage is more powerful than many administrative measures, and it is conducive to the rational allocation of groundwater resources, the sustainable utilization of groundwater resources, and the protection of the ecological environment.

By reflecting the value of groundwater as a scarce resource, the groundwater rights price can fully adjust the relationship between supply and demand of groundwater resources. When the price of water supply is raised to a certain extent, it can form a reasonable price relative to the price used for sewage treatment and regeneration, and then form an effective incentive mechanism for sewerage recycling, so as to increase the amount of available water and reduce the dependence on external water resources. Therefore, it is necessary to propose relevant policies to improve the current water resource pricing policy in Ningxia.

First, central government should amend and improve laws and policies related to water rights, making principles and methods of water rights pricing to abide by. Second, by introducing a water rights system, the government should construct a water resources allocation mode combining administrative means with market means. Third, the government should push forward water price reform step by step. The government should decentralize the power of pricing step by step, set the water price according to different regions and uses, and gradually raise current water price standards without arousing opposition from the people to ensure that water resources prices conform to market rules. Last, the government should strengthen the supervision and management of water price policy implementation. Without strict and effective supervision, no matter how good the policy is, it will not work. This would help the realization of sustainable development and utilization of water resources and increase public awareness of the need for water savings and could improve water use efficiency.

The main innovations of this study are summarized as follows: First, this study has proposed a groundwater rights pricing method combining natural, economic, and social factors, which comprehensively reflects the economic and social attributes of groundwater resources and aligns better with reality. Second, this study has calculated the price of groundwater rights in Ningxia from 2013 to 2017, and pointed out that the current water price needs to be improved. Third, it puts forward specific suggestions for water resources governance and water price reform in Ningxia, which might be favorable four saving groundwater resources and the sustainable development of water resources in China.

This paper highlights a number of areas for further research. First, the indicator system should be refined. The factors influencing groundwater rights pricing reflect a complex system integrating natural, social, and economic factors. This inevitably leads to the simplification of the indicator system. Second, when calculating the affordability of water price, this study only accounted for urban residents, and did not consider agricultural, industrial, and ecological water use. Further study is needed to calculate groundwater rights pricing for these uses, based on a comprehensive consideration of industry characteristics and influencing factors. Third, future study is needed on the transaction pricing of groundwater rights among water users. The paper informs the pricing of groundwater rights by government departments, based on groundwater value. Scientifically and rationally allocating groundwater resources requires the opening of the water rights market. Establishing methods for conducting the transaction and determining the transaction price for water users with groundwater rights is a future research focus. Last, differential pricing according to regions and uses can be calculated based on this study. 

## Figures and Tables

**Table 1:** Groundwater Resources in Ningxia Zoning by Basin Area for 2017.

River Basin	Unit	Groundwater Resources in Mountainous Areas	Groundwater Resources in Plain Areas				Double Counting between Mountainous Areas and Plain Areas	Groundwater Resources in the Subregion
			Precipitation Recharge	Surface Water Recharge	Mountain Lateral Infiltration Recharge	Total		
Areas Irrigated by the Yellow River	10^8^ m^3^	0	0.768	14.487	0.038	15.293	0.038	15.255
Zuli River	10^8^ m^3^	0.033	0	0	0	0	0	0.033
Qingshui River	10^8^ m^3^	0.857	0	0	0	0	0	0.857
Hongliugou River	10^8^ m^3^	0.028	0	0	0	0	0	0.028
Kushui River	10^8^ m^3^	0.066	0	0	0	0	0	0.066
The Huangyou Area	10^8^ m^3^	0.046	0	0	0	0	0	0.046
The Huangzuo Area	10^8^ m^3^	0.934	0.086	0	0.764	0.850	0.764	1.020
Hulu River	10^8^ m^3^	0.359	0	0	0	0	0	0.356
Jinghe River	10^8^ m^3^	1.667	0	0	0	0	0	1.667
Endorheic Drainage Area in Yanchi	10^8^ m^3^	0	0	0	0	0	0	0
Total	10^8^ m^3^	3.990	0.854	14.487	0.802	16.143	0.802	19.331

**Table 2:** The sources of the indicators.

Ningxia Water Resources Bulletin 2013–2017 [48]; Monthly Report of Groundwater Regime in China January 2013–December 2017” [51]	Groundwater quality grade, average depth of shallow groundwater, the proportion of the groundwater supply, per capita groundwater withdrawal, agricultural water withdrawal, industrial water withdrawal, withdrawal for residential living use, and the proportion of overexploited groundwater area
Ningxia Statistical Yearbook (2013–2017) [49]; China Tap Water Price 2013–2017” [52]	Population density, per capita groundwater resources, groundwater resources per unit area, and wastewater discharge
Ningxia Autonomous Region Statistical Bulletin of National Economic and Social Development (2013–2017) [50]	Per capita GDP and per 10^4^-yuan-GDP water consumption

**Table 3:** Groundwater rights value evaluation indicators for 2013–2017.

Cluster	Evaluating Indicator	Unit	Definition	Actual Number				
				2013	2014	2015	2016	2017
Environment	Groundwater Quality Grade	/	Characterizes the quality of groundwater	III	III	III	III	III
	Per Capita Groundwater Resources	m^3^	Characterizes the scarcity of groundwater resources per capita	338.30	321.99	312.60	275.13	283.45
	Groundwater Resources Per Unit Area	10^2^ m^3^/ km^2^	Characterizes the scarcity of groundwater resources regionally	427	412	403	359	373
	Average Depth of Shallow Groundwater	m	Characterizes the accessibility of groundwater	2.34	2.26	2.29	2.30	2.41
	Proportion of Overexploited Groundwater Area	%	Characterizes the severity groundwater overexploitation	1.43	1.43	1.43	1.43	1.43
Society	Population Density	people/km^2^	Characterizes the level of local population size	1253	1295	1336	1343	1388
	Per Capita Groundwater Withdrawal	m^3^	Characterizes the level of groundwater demand	85.0	82.7	76.9	78.6	81.1
	Agricultural Groundwater Withdrawal	10^8^ m^3^	Characterizes the level of agricultural groundwater consumption	1.168	1.142	1.253	1.296	1.567
	Industrial Groundwater Withdrawal	10^8^ m^3^	Characterizes the level of industrial groundwater consumption	2.594	2.426	1.847	1.638	1.436
	Residential Groundwater Use Withdrawal	10^8^ m^3^	Characterizes the level of groundwater consumption in social life	1.295	1.387	1.500	1.916	2.077
Economy	Per Capita GDP	10^3^ yuan	Characterizes the regional economic level	39.6	41.8	43.8	47.2	50.8
	Per 10^4^-yuan-GDP Water Consumption	m^3^	Characterizes water use efficiency of regional economic sectors	311	281	260	206	194
	Groundwater Supply Proportion	%	Characterizes the degree of reliance on groundwater	7.7	7.8	7.3	8.2	8.4
	Wastewater Discharge	10^7^ ton	Characterizes the negative externality of economy	38.5	37.3	32.0	33.9	30.7

**Table 4:** Evaluation criteria of groundwater rights value.

Cluster	Evaluating Indicator	Unit	Character	Evaluation Criteria				
				High	Relatively High	Common	Relatively Low	Low
Environment	Groundwater Quality Grade	/	+	>II	III	IV	V	<V
	Per Capita Groundwater Resources	m^3^	-	100.00	450.00	800.00	1150.00	1500.00
	Groundwater Resources Per Unit Area	10^2^ m^3^/ km^2^	-	200	900	1600	2300	3000
	Average Depth of Shallow Groundwater	m	+	30.00	17.25	15.50	8.25	1.00
	Proportion of Overexploited Groundwater Area	%	+	10.00	7.50	5.00	2.50	0.00
Society	Population Density	people/km^2^	+	4500	3700	2900	2100	1300
	Per Capita Groundwater Withdrawal	m^3^	+	200.0	150.0	100.0	50.0	0.0
	Agricultural Groundwater Withdrawal	10^8^ m^3^	+	100.000	75.500	51.000	26.500	2.000
	Industrial Groundwater Withdrawal	10^8^ m^3^	+	17.000	12.250	8.500	4.250	0.000
	Groundwater for Residential Living Withdrawal	10^8^ m^3^	+	10.000	7.500	5.000	2.500	0.000
Economy	Per Capita GDP	10^3^ yuan	+	100.0	82.5	65.0	47.5	30.0
	Per 10^4^-yuan-GDP Water Consumption	m^3^	+	200	155	110	65	20
	Groundwater Supply Proportion	%	+	50.0	38.0	26.0	14.0	2.0
	Wastewater Discharge	10^7^ ton	+	400.0	325.0	250.0	175.0	100.0

**Table 5:** Indicator weight of groundwater rights value.

Cluster	Evaluating Indicator	Subjective Weight	Objective Weight
Environment	Groundwater Quality Grade	0.0235	0.0736
	Per Capita Groundwater Resources	0.2515	0.0697
	Groundwater Resources Per Unit Area	0.1977	0.0697
	Average Depth of Shallow Groundwater	0.0145	0.0704
	Proportion of Overexploited Groundwater Area	0.0099	0.0736
Society	Population Density	0.1281	0.0698
	Per Capita Groundwater Withdrawal	0.0178	0.0730
	Agricultural Groundwater Withdrawal	0.0492	0.0693
	Industrial Groundwater Withdrawal	0.0258	0.0740
	Groundwater for Residential Living Withdrawal	0.0055	0.0692
Economy	Per Capita GDP	0.1724	0.0695
	Per 10^4^-yuan-GDP Water Consumption	0.0717	0.0740
	Groundwater Supply Proportion	0.0042	0.0705
	Wastewater Discharge	0.0175	0.0738

**Table 6:** Indicators of groundwater rights pricing.

Indicator	Unit	2013	2014	2015	2016	2017
Per Capita Disposable Income	yuan	14,565.78	15,906.78	17,329.06	18,832.28	20,561.66
Per Capita Water Consumption of Residents	m^3^	26.28	28.25	29.82	32.59	33.73
Residents’ Tap Water Price ^1^	yuan/ m^3^	2.06	2.06	2.06	2.06	2.31
Sewage Treatment Fee	yuan/ m^3^	0.85	0.85	0.85	0.85	0.85
Water Resources Fee	yuan/ m^3^	0.31	0.41	0.57	0.72	0.72
Water Supply Cost and Normal Profit	yuan/ m^3^	0.90	0.80	0.64	0.49	0.74
Per Capita Water Fee Expenditure	yuan	54.14	58.20	61.42	67.13	77.92
Per Capita Real Disposable Income	yuan	14,086.83	15,610.19	17,140.51	18,8553.97	20,237.85
Water Rate Affordability Indicator	%	2	2	2	2	2
Ratio of Residents’ Water Fee Expenditure to Real Disposable Income	%	0.38	0.37	0.36	0.36	0.39
Groundwater Rights Price	yuan/ m^3^	5.11	5.21	5.44	5.40	5.73

^1^ There is no uniform water price standard in Ningxia. These data are based on the water pricing of major cities in the district.
